# Editorial: Causation and Causal Explanation in Psychiatry—Beyond Scientism and Skepticism

**DOI:** 10.3389/fpsyt.2017.00070

**Published:** 2017-05-09

**Authors:** Annemarie Kalis, Derek Strijbos, Leon de Bruin, Gerrit Glas

**Affiliations:** ^1^Utrecht University, Utrecht, Netherlands; ^2^Radboud University, Nijmegen, Netherlands; ^3^VU University, Amsterdam, Netherlands

**Keywords:** psychiatry, causal processes, integration, complexity, mental states

**The Editorial on the Research Topic**

**Causation and Causal Explanation in Psychiatry—Beyond Scientism and Skepticism**

Since psychiatry firmly established itself as a scientific discipline, it has been propelled forward by the hope that the different diagnostic categories distinguished in clinical practice, will turn out to correspond to unique underlying causes. However, so far there is little evidence that disorders such as major depression or schizophrenia can be traced back to relatively simple, common causal trajectories. Rather, the etiology of almost all mental disorders seems to be complex and multifactorial and to span different levels of explanation, ranging from (epi)genetic, neurobiological to psychological, and social levels.

Clinicians, broadly speaking, tend to be skeptical about the prospects of causal modeling in psychiatry, whereas scientists tend to cling to a scientistic and sometimes also reductionistic view on mental disorder. Psychiatry needs to find a way beyond skepticism and scientism, and this requires new methods and new conceptual approaches that enable us to gain a better insight into the complexity of the causal processes leading to mental disorders.

This Research Topic discusses novel theoretical and empirical strategies addressing causation and causal explanation in psychiatry, in the context of a broader discussion of what science can and cannot contribute to the definition of mental disorder. Questions addressed are: how could the complexity of mental disorders be modeled and empirically investigated? Are traditional nomological theories of causation the best framework for thinking about causation in psychiatry, or should we look at alternatives such as mechanism-based, interventionist, or pluralist theories of causation? How to integrate different levels of explanation in etiological models of mental disorder?

Dijkstra and de Bruin investigate to what extent it is justified to draw conclusions about causal relations between brain states and mental states from “traditional” cognitive neuroscience studies and brain stimulation studies. They argue that, depending on whether one adopts Woodward’s or Baumgartner’s interventionist account of causation, it is possible to draw causal conclusions from both types of studies (Woodward) or from brain stimulation studies only (Baumgartner). Also, they show what happens to these conclusions if we adopt different views of the relation between mental states and brain states.

Gijsbers reviews recent debates about the unity of science and explanatory pluralism, focusing on the tension between the integrative and the isolationist perspective: should the integrative tendencies in science be fully indulged in, or is a certain amount of isolation necessary? He argues that an important question is whether two true explanations of the same fact can ever fail to be combinable into one single explanation and shows that this can be the case when explanations have incompatible counterfactual consequences. He thus concludes that although interdisciplinarity may have many advantages, we should not take the project of integration too far.

According to Hutto, philosophy of psychiatry faces a tough choice between two competing ways of understanding mental disorders. The folk psychology (FP) view puts our everyday normative conceptual scheme in the driver’s seat. Opposing this, the scientific image (SI) view holds that our understanding of mental disorders must come from the mind sciences. This paper rejects both the FP view (in its pure form) and the SI view, in its popular cognitivist renderings. It concludes that a more liberal version of SI can accommodate what is best in both views and provide a sound philosophical basis for a future psychiatry.

Thornton focuses on the idea that psychiatry contains, in principle, a series of levels of explanation—an idea that has been criticized as presupposing a discredited pre-Humean view of causation. These claims echo some superficially similar remarks in Wittgenstein’s Zettel. Thornton argues that attention to the context of Wittgenstein’s remarks suggests a reason to reject explanatory minimalism in psychiatry and reinstate a Wittgensteinian notion of levels of explanation.

Van Riel starts from the common assumption that social environment and cultural formation shape mental disorders. The details of this claim are, however, not well understood. His paper takes a look at the claim that culture has an impact on psychiatry from the perspective of metaphysics and the philosophy of science. Its aim is to offer, in a general fashion, partial explications of some significant versions of the thesis that culture and social environment shape mental disorders and to highlight some of the consequences social constructionism about psychiatry has for psychiatric explanation.

Stein and Illes discuss the emergent field of global mental health, which has paid particular attention to upstream causal factors, for example, poverty, inequality, and gender discrimination in the pathogenesis of mental disorders. However, this field has also been criticized for relying erroneously on Western paradigms of mental illness. The authors argue that it is important to steer a path between scientism (disorders as essential categories) and skepticism (disorders as mere social constructions) and propose an integrative model that emphasizes the contribution of a broad range of causal mechanisms and the consequent importance of broad spectrum approaches to intervention.

Young presents a hybrid top-down, bottom-up model of the relationship between symptoms and mental disorder, viewing symptom expression and their causal complex as a reciprocally dynamic system with multiple levels, from lower-order symptoms in interaction to higher-order constructs affecting them. He concludes that symptoms vary over several dimensions, including: subjectivity, objectivity, conscious motivation effort, and unconscious influences, and discusses the degree to which individual (e.g., meaning) and universal (e.g., causal) processes are involved.

Bechtel reviews some of the compelling evidence of disrupted circadian rhythms in individuals with mood disorders (major depressive disorder, seasonal affective disorder, and bipolar disorder). While the evidence is suggestive of an etiological role for altered circadian rhythms in mood disorders, it is compatible with other explanations. In light of this, the paper advances a proposal as to what evidence would be needed to establish a direct causal link between disruption of circadian rhythms and mood disorders.

Bielczyk et al. integrate the literature on cognitive and physiological biomarkers of MDD with the insights derived from mathematical models of brain networks. They propose a new approach called “circuit to construct mapping,” which aims to characterize causal relations between the underlying network dynamics (as the cause) and the constructs referring to the clinical symptoms of MDD (as the effect).

## Author Contributions

AK, DS, LB, and GG wrote and approved the manuscript.

## Conflict of Interest Statement

The authors declare that the research was conducted in the absence of any commercial or financial relationships that could be construed as a potential conflict of interest.

